# Supplementary material for: Parental, pregnancy and neonatal characteristics during the perinatal period as potential risk factors for childhood cancer: FeToxCancer case-control study
Source: PLoS One. 2026 Apr 16;21(4):e0333752. doi: 10.1371/journal.pone.0333752 (PMC13086354; doi:10.1371/journal.pone.0333752)
Supplement: S1 Table — (DOCX) [file pone.0333752.s001.docx]

S1 Table. Distribution of studied perinatal characteristics between cases of leukaemia, CNS tumours, lymphoma and other cancer types combined and matching controls.

| **Perinatal characteristics** | **Leukaemia (n=345)** | | **CNS tumour (n =328)** | | **Lymphoma (n =153)** | | **Other cancer**  **types combined (n =517)** | |
| --- | --- | --- | --- | --- | --- | --- | --- | --- |
|  | Cases  N(%^a^) | Controls  N(%^a^) | Cases  N(%^a^) | Controls  N(%^a^) | Cases  N(%^a^) | Controls  N(%^a^) | Cases  N(%^a^) | Controls  N(%^a^) |
| **Maternal cancer** |  |  |  |  |  |  |  |  |
| No | 303 (88) | 3042 (8) | 285 (87) | 2853 (87) | 132 (86) | 1334 (87) | 440 (85) | 4453 (86) |
| Yes | 42 (12) | 408 (12) | 43 (13) | 427 (13) | 21 (14) | 196 (13) | 77 (15) | 717 (14) |
| **Maternal age (years)** |  |  |  |  |  |  |  |  |
| <25 | 58 (17) | 640 (19) | 52 (16) | 615 (19) | 31 (20) | 261 (17) | 89 (17) | 912 (18) |
| 25-34 | 228 (66) | 2241 (65) | 222 (68) | 2099 (64) | 103 (67) | 1027 (67) | 339 (66) | 3504 (68) |
| ≥35 | 59 (17) | 569 (16) | 54 (16) | 566 (17) | 19 (12) | 242 (16) | 89 (17) | 754 (15) |
| **Paternal age (years)** |  |  |  |  |  |  |  |  |
| <25 | 23 (7) | 248 (7) | 23 (7) | 222 (7) | 15 (10) | 103 (7) | 35 (7) | 343 (7) |
| 25-34 | 190 (56) | 1951 (57) | 186 (57) | 1880 (58) | 101 (66) | 925 (61) | 280 (55) | 3026 (59) |
| ≥35 | 129 (38) | 1220(36) | 117 (36) | 1155 (35) | 36 (24) | 493 (32) | 196 (38) | 1753 (34) |
| missing | 3 (0.9) | 31 (0.9) | 2 (0.6) | 23 (0.7) | 1 (0.6) | 9 (0.6) | 6 (1.1) | 48 (0.9) |
| **Maternal education** |  |  |  |  |  |  |  |  |
| Primary | 29 (8) | 323 (9) | 31 (10) | 327 (10) | 12 (8) | 153 (10) | 48 (9) | 518 (10) |
| Secondary | 153 (45) | 1391 (41) | 133 (41) | 1396 (43) | 67 (44) | 640 (42) | 215 (42) | 2162 (42) |
| Postsecondary | 161 (47) | 1699 (50) | 161 (49) | 1530 (47) | 74 (48) | 722 (48) | 253 (49) | 2447 (48) |
| missing | 2 (0.6) | 37 (1) | 3 (0.9) | 27 (0.8) | - | 15 (0.9) | 1 (0.2) | 43 (0.8) |
| **Paternal education** |  |  |  |  |  |  |  |  |
| Primary | 41 (12) | 472 (14) | 44 (14) | 465 (14) | 22 (15) | 203 (13) | 78 (15) | 696 (14) |
| Secondary | 180 (53) | 1628 (49) | 15 (48) | 1608 (50) | 72 (48) | 790 (52) | 245 (48) | 2558 (50) |
| Postsecondary | 121 (35) | 1286 (38) | 124 (38) | 1147 (36) | 57 (38) | 518 (34) | 186 (509) | 1821 (36) |
| Missing | 3 (0.9) | 64 (2) | 2 (0.6) | 60 (2) | 2 (1) | 19 (1) | 8 (2) | 95 (2) |
| **Parity** |  |  |  |  |  |  |  |  |
| 1 | 138 (40) | 1494 (43) | 149 (45) | 1406 (43) | 71 (46) | 673 (44) | 216 (42) | 2262 (44) |
| 2 | 130 (38) | 1270 (37) | 119 (36) | 1169 (36) | 52 (34) | 563 (37) | 187 (36) | 1835 (36) |
| ≥3 | 77 (22) | 686 (20) | 60 (18) | 705 (21) | 30 (20) | 294 (19) | 114 (22) | 1055 (20) |
| **Maternal BMI (kg/m^2^)^b^** |  |  |  |  |  |  |  |  |
| <18.5 | 15 (5) | 63 (2) | 5 (2) | 62 (2) | 1 (0.8) | 36 (3) | 13 (3) | 133 (3) |
| 18.5–24.9 | 171 (57) | 1833 (62) | 168 (61) | 1735 (63) | 68 (55) | 798 (64) | 272 (64) | 2671 (62) |
| 25–29.9 | 84 (28) | 727 (24) | 68 (25) | 673 (24) | 34 (27) | 308 (25) | 99 (23) | 1020 (24) |
| ≥30 | 20 (10) | 345 (12) | 34 (12) | 282 (10) | 21 (17) | 106 (8) | 42 (10) | 457 (11) |
| missing | 45 (13) | 482 (14) | 53 (16) | 528 (16) | 29 (19) | 282 (19) | 89 (17) | 889 (17) |
| **Maternal smoking^b^** |  |  |  |  |  |  |  |  |
| No | 287 (85) | 1899 (87) | 273 (87) | 2728 (86) | 121 (86) | 1253 (84) | 420 (86) | 4227 (85) |
| Yes | 49 (15) | 429 (13) | 42 (13) | 458 (14) | 20 (14) | 232 (16) | 71 (14) | 749 (15) |
| missing | 9 (3) | 122 (3) | 13 (4) | 94 (3) | 12 (8) | 45 (3) | 26 (5) | 194 (4) |
| **Assisted pregnancy IVF** |  |  |  |  |  |  |  |  |
| No | 337 (97.7) | 3371 (97.7) | 323 (98.5) | 3215 (98) | 148 (97) | 1502 (98) | 509 (98.4) | 5050 (97.7) |
| Yes | 8 (2.3) | 79 (2.3) | 5 (1.5) | 65 (2) | 5 (3) | 28 (2) | 8 (1.6) | 120 (2.3) |
| **Mode of delivery** |  |  |  |  |  |  |  |  |
| Vaginal | 276 (80) | 2785 (81) | 261 (80) | 2642 (81) | 124 (81) | 1249 (82) | 410 (79) | 4242 (82) |
| caesarean elective | 16 (5) | 201 (6) | 15 (5) | 178 (5) | 6 (4) | 71 (5) | 36 (7) | 265 (5) |
| caesarean emergency | 35 (10) | 267 (8) | 29 (9) | 269 (8) | 12 (8) | 117 (8) | 46 (9) | 395 (8) |
| forceps or vacuum | 18 (5) | 197 (6) | 23 (7) | 191 (6) | 11 (7) | 93 (6) | 25 (5) | 268 (5) |
| **Child infection^d^** |  |  |  |  |  |  |  |  |
| No | 323 (96) | 3269 (97) | 307 (97) | 3154 (96) | 140 (96) | 1479 (97) | 480 (97) | 4767 (97) |
| Yes | 14 (4) | 101 (3) | 9 (3) | 126 (4) | 6 (4) | 51 (3) | 13 (3) | 163 (3) |
| missing | 8 (2) | 80 (2) | 12 (4) | 120 (4) | 7 (4) | 70 (4) | 24 (5) | 240 (5) |
| **GA (weeks)** |  |  |  |  |  |  |  |  |
| <37 | 20 (6) | 196 (6) | 29 (9) | 206 (6) | 10 (7) | 98 (6) | 41 (8) | 334 (6) |
| 37 – 41 | 305 (88) | 3037 (88) | 280 (85) | 2856 (87) | 130 (85) | 1323 (86) | 445 (86) | 4494 (87) |
| ≥42 | 20 (6) | 217 (6) | 19 (6) | 218 (7) | 13 (9) | 109 (7) | 31 (6) | 342 (7) |
| **Birthweight for GA**^c^ |  |  |  |  |  |  |  |  |
| AGA | 304 (89) | 3153 (92) | 297 (91) | 3004 (92) | 138 (90.8) | 1395 (91.5) | 464 (90) | 4701 (91) |
| SGA | 15 (4) | 141 (4) | 14 (4.3) | 126 (4) | 4 (2.6) | 54 (3.5) | 23 (4.5) | 221 (4.2) |
| LGA | 24 (7) | 145 (4) | 14 (4.3) | 143 (4.4) | 10 (6.6) | 76 (5.0) | 28 (5) | 228 (4.4) |
| Missing | 2 (0.6) | 11 (0.3) | 3 (0.9) | 7 (0.2) | 1 (0.6) | 5 (0.3) | 5 (0.9) | 20 (0.4) |
| **5min-Apgar** |  |  |  |  |  |  |  |  |
| ≥7 | 336 (98) | 3400 (99) | 323 (99.4) | 3234 (99) | 150 (98) | 1493 (98.2) | 501 (97) | 5098 (99) |
| <7 | 7 (2) | 36 (1) | 2 (0.6) | 32 (1) | 3 (2) | 27 (1.8) | 13 (3) | 54 (1) |
| missing | 2 (0.5) | 14 (0.4) | 3 (0.9) | 14 (0.4) | - | 10 (0.6) | 3 (0.5) | 18 (0.3) |
| **Neonatal care^e^** |  |  |  |  |  |  |  |  |
| No | 240 (88) | 2486 (90) | 226(87) | 2327 (89) | 94 (86) | 984 (90) | 312 (84) | 3308 (89) |
| Yes | 34 (12) | 254 (10) | 35 (13) | 283 (11) | 15 (14) | 106 (10) | 61 (16) | 422 (11) |
| missing | 71 (20) | 710 (20) | 67 (20) | 670 (20) | 44 (29) | 440 (29) | 144 (28) | 1440 (28) |

^a^ – based on non-missing data; ^b^ – maternal body mass index (BMI, kg/m2) and smoking status at enrolment into maternal health care; IVF – in vitro fertility; ^c^–based on weight, sex , GA and calculated according to Marsál et al., 1996;

^d^ – any infection during pregnancy; ^e^– data available since 2002
