# Supplementary material for: Parental, pregnancy and neonatal characteristics during the perinatal period as potential risk factors for childhood cancer: FeToxCancer case-control study
Source: PLoS One. 2026 Apr 16;21(4):e0333752. doi: 10.1371/journal.pone.0333752 (PMC13086354; doi:10.1371/journal.pone.0333752)
Supplement: S2 Table — (DOCX) [file pone.0333752.s002.docx]

S2 Table. Associations of perinatal characteristics with leukaemia

| **Perinatal characteristics** | Crude HR (95%CI) | Crude HR (95%CI)  Complete data^a^ | **Model 1**  HR (95%CI) | **Model 2**  HR (95%CI) | **Model 3**  HR (95%CI) |  |
| --- | --- | --- | --- | --- | --- | --- |
| **Parental characteristics** | | | | | | |
| **Maternal cancer, N** | 3795/345 | 3124/291 | 3124/291 | 3124/291 | 3124/291 |  |
| No | ref |  | Ref | Ref | Ref |  |
| Yes | 0.98 (0.71, 1.34) | 1.01 (0.71, 1.44) | 1.02 (0.72, 1.45) | 1.03 (0.72, 1.47) | 1.03 (0.72, 1.47) |  |
| **Maternal age (years), N** | 3795/345 | 3124/291 | 3124/291 | 3124/291 | 3124/291 |  |
| <25 | ref | Ref | Ref | Ref | Ref |  |
| 25-34 | 1.13 (0.84, 1.50) | 1.17 (0.84, 1.62) | 1.10 (0.75, 1.60) | 1.08 (0.74, 1.58) | 1.08 (0.74, 1.58) |  |
| ≥35 | 1.20 (0.83, 1.73) | 1.30 (0.87, 1.94) | 1.17 (0.71, 1.90) | 1.15 (0.70, 1.89) | 1.15 (0.70, 1.89) |  |
| **Paternal age (years), N** | 3761/342 | 3124/291 | 3124/291 | 3124/291 | 3124/291 |  |
| <25 | Ref | Ref | Ref | Ref | Ref |  |
| 25-34 | 1.08 (0.69, 1.66) | 1.39 (0.81, 2.36) | 1.36 (0.76, 2.42) | 1.35 (0.76, 2.42) | 1.36 (0.76, 2.43) |  |
| ≥35 | 1.19 (0.76, 1.85) | 1.56 (0.90, 2.67) | 1.49 (0.80, 2.79) | 1.50 (0.80, 2.80) | 1.51 (0.81, 2.81) |  |
| **Maternal education, N** | 3756 /343 | 3124/291 | 3124/291 | 3124/291 | 3124/291 |  |
| Primary | ref | Ref | Ref | Ref | Ref |  |
| Secondary | 1.19 (0.80, 1.78) | 0.95 (0.63, 1.45) | 0.94 (0.61, 1.44) | 0.95 (0.62, 1.45) | 0.95 (0.62, 1.45) |  |
| Postsecondary | 1.08 (0.72, 1.60) | 0.92 (0.60, 1.38) | 0.91 (0.58, 1.43) | 0.92 (0.58, 1.45) | 0.93 (0.59, 1.45) |  |
| **Paternal education, N** | 3728/342 | 3124/291 | 3124/291 | 3124/291 | 3124/291 |  |
| Primary | Ref | Ref | Ref | Ref | Ref |  |
| Secondary | 1.20 (0.80, 1.78) | 1.10 (0.77, 1.56) | 1.12 (0.78, 1.62) | 1.12 (0.78, 1.61) | 1.11 (0.77, 1.60) |  |
| Postsecondary | 1.08 (0.72, 1.60) | 0.94 (0.65, 1.36) | 0.93 (0.62, 1.40) | 0.94 (0.62, 1.41) | 0.93 (0.61, 1.40) |  |
| **Parity, N** | 3795/345 | 3124/291 | 3124/291 | 3124/291 | 3124/291 |  |
| 1 | Ref | Ref | Ref | Ref | Ref |  |
| 2 | 1.27 (0.90, 1.78) | 1.22 (0.95, 1.58) | 1.16 (0.89, 1.51) | 1.19 (0.90, 1.56) | 1.19 (0.90, 1.56) |  |
| ≥3 | 1.12 (0.78, 1.59) | 1.20 (0.88, 1.63) | 1.05 (0.73, 1.48) | 1.09 (0.77, 1.55) | 1.09 (0.77, 1.55) |  |
| **Maternal BMI (kg/m^2^)^b^, N** | 3268/ 300 | 3124/291 | 3124/291 | 3124/291 | 3124/291 |  |
| <18.5 | **2.34 (1.38, 3.97)**** | **2.32 (1.34, 4.00)**** | **2.44 (1.41, 4.23)**** | **2.44 (1.41, 4.23)**** | **2.43 (1.40, 4.22)**** |  |
| 18.5–24.9 | ref | ref | Ref | Ref | Ref |  |
| 25–29.9 | 1.24 (0.96, 1.62) | 1.28 (0.98, 1.66) | 1.24 (0.94, 1.62) | 1.23 (0.94, 1.61) | 1.23 (0.94, 1.61) |  |
| ≥30 | 0.95 (0.64, 1.41) | 0.97 (0.65, 1.44) | 0.94 (0.63, 1.40) | 0.92 (0.62, 1.38) | 0.92 (0.62, 1.38) |  |
| **Maternal smoking^b^, N** | 3268/300 | 3124/291 | 3124/291 | 3124/291 | 3124/291 |  |
| No | Ref | Ref | Ref | Ref | Ref |  |
| Yes | 1.07 (0.78, 1.44) | 1.04 (0.73, 1.46) | 1.03 (0.72, 1.47) | 1.02 (0.71, 1.47) | 1.03 (0.72, 1.47) |  |
| **Pregnancy characteristics** | | | | | | |
| **Assisted pregnancy IVF, N** | 3795/345 | 3124/291 | 3124/291 | 3124/291 | 3124/291 |  |
| No | Ref | Ref | Ref | Ref | Ref |  |
| Yes | 1.08 (0.53, 2.17) | 1.02 (0.48, 2.16) | 1.03 (0.48, 2.23) | 1.03 (0.47, 2.21) | 1.06 (0.49, 2.29) |  |
| **Mode of delivery, N** | 3795/345 | 3124/291 | 3124/291 | 3124/291 | 3124/291 |  |
| Vaginal no instruments | Ref | Ref | Ref | Ref | Ref |  |
| caesarean elective | 0.85 (0.51, 1.40) | 0.88 (0.52, 1.52) | 0.86 (0.50, 1.48) | 0.86 (0.50, 1.48) | 0.86 (0.50, 1.48) |  |
| caesarean emergency | 1.33 (0.94, 1.90) | 1.19 (0.79, 1.78) | 1.22 (0.81, 1.85) | 1.23 (0.81, 1.85) | 1.28 (0.84, 1.94) |  |
| forceps or vacuum | 0.93 (0.58, 1.51) | 1.03 (0.63, 1.69) | 1.11 (0.67, 1.84) | 1.11 (0.67, 1.84) | 1.12 (0.67, 1.85) |  |
| **Neonatal characteristics** | | | | | | |
| **GA (weeks), N** | 3795/345 | 3124/291 | 3124/291 | 3124/291 | 3124/291 |  |
| <37 | 1.00 (0.64, 1.59) | 0.85 (0.49, 1.48) | 0.85 (0.48, 1.49) | 0.80 (0.45, 1.42) | 0.80 (0.45, 1.42) |  |
| 37 – 41 | Ref | Ref | Ref | Ref | Ref |  |
| ≥42 | 0.90 (0.57, 1.41) | 0.90 (0.55, 1.47) | 0.94 (0.57, 1.54) | 0.92 (0.56, 1.50) | 0.92 (0.56, 1.50) |  |
| **Birthweight for GA**^c^**, N** | 3782/343 | 3114/290 | 3114/290 | 3114/290 | 3114/290 |  |
| AGA | Ref | Ref | Ref | Ref | Ref |  |
| SGA | 1.07 (0.63, 1.79) | 0.97 (0.54, 1.73) | 0.96 (0.53, 1.72) | 0.93 (0.51, 1.68) | 0.94 (0.52, 1.72) |  |
| LGA | **1.66 (1.10, 2.51)*** | **1.56 (1.01, 2.46)*** | **1.58 (1.02, 2.51)*** | **1.59 (1.01, 2.52)*** | **1.58 (1.01, 2.51)*** |  |
| **Child infection-I**^d^**, N** | 3707/337 | 3052/285 | 3052/285 | 3052/285 | 3052/285 |  |
| No | Ref | Ref | Ref | Ref | Ref |  |
| Yes | 1.33 (0.78, 2.27) | 1.36 (0.74, 2.49) | 1.39 (0.76, 2.55) | 1.39 (0.76, 2.56) | 1.39 (0.75, 1.55) |  |
| **5-min Apgar, N** | 3779/343 | 3113 /289 | 3113 /289 | 3113 /289 | 3113 /289 |  |
| ≥7 | Ref | Ref | Ref | Ref | Ref |  |
| <7 | *NA* | *NA* | *NA* | *NA* | *NA* |  |
| **Neonatal care^e^, N** | 3014/274 | 2681/249 | 2681/249 | 2681/249 | 2681/249 |  |
| No | Ref | Ref | Ref | Ref | Ref |  |
| Yes | 1.34 (0.94, 1.92) | 1.22 (0.81, 1.82) | 1.18 (0.79, 1.77) | 1.16 (0.76, 1.76) | 1.27 (0.82, 1.98) |  |

N, n of total observations/n of events, GA – gestational age; IVF – in vitro fertilisation; BMI – body mass index; AGA – adequate for GA, SGA- small for GA, LGA - large for GA; NA – less than 10 observations.

*** p < 0.001, ** p < 0.01, * p < 0.05; models 1 to 3 – shaded are perinatal characteristics used as adjustment covariates in the respective model.

^a^ – according to complete data for all used adjustment covariates; ^b^– smoking and BMI at the time of enrolment into the maternal health care; ^c^– calculated according to birthweight, sex and gestational age; ^d^ – data according to the incoming patient registry; ^e^ – data available since 1995
