# Supplementary material for: Parental, pregnancy and neonatal characteristics during the perinatal period as potential risk factors for childhood cancer: FeToxCancer case-control study
Source: PLoS One. 2026 Apr 16;21(4):e0333752. doi: 10.1371/journal.pone.0333752 (PMC13086354; doi:10.1371/journal.pone.0333752)
Supplement: S3 Table — (DOCX) [file pone.0333752.s003.docx]

S3 Table. Associations of perinatal characteristics with CNS tumours.

| **Perinatal characteristics** | Crude HR (95%CI) | Crude HR (95%CI)  Complete data^a^ | **Model 1**  HR (95%CI) | **Model 2**  HR (95%CI) | **Model 3**  HR (95%CI) |  |
| --- | --- | --- | --- | --- | --- | --- |
| **Parental characteristics** | | | | | | |
| **Maternal cancer, N** | 3608 /328 | 2911/ 264 | 2911/ 264 | 2911/ 264 | 2911/ 264 |  |
| No | ref | Ref | Ref | Ref | Ref |  |
| Yes | 0.94 (0.68, 1.30 | 0.88 (0.61, 1.27) | 0.87 (0.60, 1.26) | 0.87 (0.60, 1.26) | 0.87 (0.60, 1.26) |  |
| **Maternal age (years) N** | 3608 /328 | 2911/ 264 | 2911/ 264 | 2911/ 264 | 2911/ 264 |  |
| <25 | ref | Ref | Ref | Ref | Ref |  |
| 25-34 | 1.25 (1.43, 1.69) | 1.29 (0.94, 1.98) | 1.47 (0.97, 2.19) | 1.48 (0.98, 2.22) | 1.48 (0.98, 2.22) |  |
| ≥35 | 1.17 (0.80, 1.71) | 1.10 (0.70, 1.72) | 1.15 (0.66, 1.98) | 1.18 (0.68, 2.05) | 1.19 (0.68, 2.06) |  |
| **Paternal age (years), N** | 3583 /326 | 2911/ 264 | 2911/ 264 | 2911/ 264 | 2911/ 264 |  |
| <25 | Ref | Ref | Ref | Ref | Ref |  |
| 25-34 | 0.96 (0.63, 1.49) | 0.96 (0.63, 1.49) | 0.85 (0.48, 1.52) | 0.86 (0.48, 1.52) | 0.85 (0.48, 1.51) |  |
| ≥35 | 1.02 (0.65, 1.59) | 1.02 (0.65, 1.59) | 0.96 (0.52, 1.79) | 0.97 (0.52, 1.80) | 0.97 (0.52, 1.80) |  |
| **Maternal education, N** | 3578 /325 | 2911/ 264 | 2911/ 264 | 2911/ 264 | 2911/ 264 |  |
| Primary | ref | Ref | Ref | Ref | Ref |  |
| Secondary | 1.00 (0.68, 1.48) | 1.02 (0.66, 1.59) | 0.88 (0.56, 1.39) | 0.89 (0.56, 1.40) | 0.88 (0.56, 1.39) |  |
| Postsecondary | 1.13 (0.77, 1.66) | 1.12 (0.72, 1.72) | 0.89 (0.55, 1.42) | 0.88 (0.54, 1.43) | 0.89 (0.55, 1.43) |  |
| **Paternal education, N** |  | 2911/ 264 | 2911/ 264 | 2911/ 264 | 2911/ 264 |  |
| Primary | Ref | Ref | Ref | Ref | Ref |  |
| Secondary | 1.01 (0.72, 1.41) | 1.31 (0.87, 1.97) | 1.30 (0.86, 1.98) | 1.30 (0.86, 1.98) | 1.30 (0.86, 1.98) |  |
| Postsecondary | 1.15 (0.82, 1.63) | 1.42 (0.93, 2.16) | 1.39 (0.89, 2.19) | 1.39 (0.89, 2.19) | 1.39 (0.89, 2.19) |  |
| **Parity, N** | 3608 /328 | 2911/ 264 | 2911/ 264 | 2911/ 264 | 2911/ 264 |  |
| 1 | Ref | Ref | Ref | Ref | Ref |  |
| 2 | 0.96 (0.76, 1.23) | 0.99 (0.76, 1.29) | 0.94 (0.71, 1.23) | 0.93 (0.70, 1.23) | 0.93 (0.70, 1.23) |  |
| ≥3 | 0.81 (0.60, 1.09) | 0.76 (0.54, 1.07) | 0.70 (0.48, 1.02) | 0.69 (0.47, 1.02) | 0.70 (0.47, 1.03) |  |
| **Maternal BMI (kg/m^2^)^b^, N** | 3027/275 | 2911/ 264 | 2911/ 264 | 2911/ 264 | 2911/ 264 |  |
| <18.5 | 0.86 (0.35, 2.09) | 0.94 (0.39, 2.29) | 0.99 (0.41, 2.43) | 0.99 (0.40, 2.42) | 0.99 (0.41, 2.43) |  |
| 18.5–24.9 | Ref | Ref | Ref | Ref | Ref |  |
| 25–29.9 | 1.09 (0.82, 1.45) | 1.15 (0.87, 1.53) | 1.19 (0.88, 1.57) | 1.18 (0.88, 1.57) | 1.17 (0.88, 1.57) |  |
| ≥30 | **1.31 (1.00, 1.99)*** | **1.41 (1.01, 2.06)*** | **1.51 (1.04, 2.21)*** | **1.52 (1.04, 2.22)*** | **1.51 (1.04, 2.21)*** |  |
| **Maternal smoking^b^, N** | 3501/315 | 2911/ 264 | 2911/ 264 | 2911/ 264 | 2911/ 264 |  |
| No | Ref | Ref | Ref | Ref | Ref |  |
| Yes | 0.85 (0.61, 1.18) | 0.86 (0.59, 1.24) | 0.98 (0.66, 1.42) | 0.96 (0.65, 1.42) | 0.96 (0.65, 1.42) |  |
| **Pregnancy characteristics** | | | | | | |
| **Assisted pregnancy IVF, N** | 3608 /328 | 2911/ 264 | 2911/ 264 | 2911/ 264 | 2911/ 264 |  |
| No | Ref | Ref | Ref | Ref | Ref |  |
| Yes | 0.84 (0.34, 2.02) | 0.78 (0.29, 2.09) | 0.72 (0.26, 1.97) | 0.73 (0.27, 1.97) | 0.72 (0.26, 1.97) |  |
| **Mode of delivery, N** | 3608 /328 | 2911/ 264 | 2911/ 264 | 2911/ 264 | 2911/ 264 |  |
| Vaginal no instruments | Ref | Ref | Ref | Ref | Ref |  |
| caesarean elective | 0.93 (0.55, 1.56) | 0.73 (0.39, 1.39) | 0.72 (0.38, 1.37) | 0.72 (0.38, 1.37) | 0.71 (0.37, 1.34) |  |
| caesarean emergency | 1.15 (0.70, 0.49) | 1.03 (0.67, 1.58) | 0.94 (0.61, 1.46) | 0.95 (0.62, 1.48) | 0.92 (0.59, 1.44) |  |
| forceps or vacuum | 1.25 (0.82, 1.92) | 1.09 (0.66, 1.82) | 1.03 (0.61, 1.74) | 1.03 (0.61, 1.74) | 1.04 (0.62, 1.75) |  |
| **Neonatal characteristics** | | | | | | |
| **GA (weeks), N** | 3608 /328 | 2911/ 264 | 2911/ 264 | 2911/ 264 | 2911/ 264 |  |
| <37 | 1.42 (0.97, 2.09) | 1.24 (0.78, 2.00) | 1.20 (0.76, 1.90) | 1.25 (0.78, 2.01) | 1.25 (0.78, 2.01) |  |
| 37 – 41 | Ref | Ref | Ref | Ref | Ref |  |
| ≥42 | 0.89 (0.56, 1.41) | 0.97 (0.58, 1.61) | 0.99 (0.60, 1.66) | 0.98 (0.59, 1.64) | 0.98 (0.59, 1.64) |  |
| **Birthweight for GA**^c^**, N** | 3598/325 | 2904/261 | 2904/261 | 2904/261 | 2904/261 |  |
| AGA | Ref | Ref | Ref | Ref | Ref |  |
| SGA | 1.17 (0.68, 2.00) | 1.06 (0.56, 2.00) | 1.07 (0.57, 2.02) | 1.12 (0.58, 2.12) | 1.10 (0.58, 2.09) |  |
| LGA | 1.00 (0.58, 1.71) | 1.13 (0.65, 1.97) | 1.10 (0.61, 1.93) | 1.12 (0.63, 1.99) | 1.11 (0.62, 1.97) |  |
| **Child infection-I**^d^**, N** | 3476 /316 | 2798/254 | 2798/254 | 2798/254 | 2798/254 |  |
| No | Ref | Ref | Ref | Ref | Ref |  |
| Yes | *NA* | *NA* | *NA* | *NA* | *NA* |  |
| **5-min Apgar, N** | 3591/325 | 2896/261 | 2896/261 | 2896/261 | 2896/261 |  |
| ≥7 | Ref | Ref | Ref | Ref | Ref |  |
| <7 | *NA* | *NA* | *NA* | *NA* | *NA* |  |
| **Neonatal care^e^, N** | 2871/261 | 2516/228 | 2516/228 | 2516/228 | 2516/228 |  |
| No | Ref | Ref | Ref | Ref | Ref |  |
| Yes | 1.27 (0.89, 1.82) | 1.17 (0.67, 1.54) | 0.98 (0.64, 1.49) | 1.00 (0.65, 1.54) | 0.88 (0.53, 1.45) |  |

N, n of total observations/n of events, GA – gestational age; IVF – in vitro fertilisation; BMI – body mass index; AGA – adequate for GA, SGA- small for GA, LGA - large for GA; NA – less than 10 observations;

*** p < 0.001, ** p < 0.01, * p < 0.05; models 1 to 3 – shaded are perinatal characteristics used as adjustment covariates in the respective model.

^a^ – according to complete data for all used adjustment covariates; ^b^– smoking and BMI at the time of enrolment into maternal health care; ^c^– calculated according to birthweight, sex and gestational age; ^d^ – data according to the incoming patient registry; ^e^ – data available since 1995
