# Supplementary material for: Parental, pregnancy and neonatal characteristics during the perinatal period as potential risk factors for childhood cancer: FeToxCancer case-control study
Source: PLoS One. 2026 Apr 16;21(4):e0333752. doi: 10.1371/journal.pone.0333752 (PMC13086354; doi:10.1371/journal.pone.0333752)
Supplement: S4 Table — (DOCX) [file pone.0333752.s004.docx]

S4 Table. Associations of perinatal characteristics with lymphoma.

| **Perinatal characteristics** | Crude HR (95%CI) | Crude HR (95%CI)  Complete data^a^ | **Model 1**  HR (95%CI) | **Model 2**  HR (95%CI) | **Model 3**  HR (95%CI) |  |
| --- | --- | --- | --- | --- | --- | --- |
| **Parents characteristics** | | | | | | |
| **Maternal cancer, N** | 1683/153 | 1322/115 | 1322/115 | 1322/115 | 1322/115 |  |
| No | ref | Ref | Ref | Ref | Ref |  |
| Yes | 1.04 (0.66, 1.65) | 1.07 (0.62, 1.83) | 1.11 (0.65, 1.92) | 1.10 (0.63, 1.89) | 1.10 (0.63, 1.89) |  |
| **Maternal age (years) , N** | 1683/153 | 1322/115 | 1322/115 | 1322/115 | 1322/115 |  |
| <25 | ref | Ref | Ref | Ref | Ref |  |
| 25-34 | 0.87 (0.58, 1.29) | 0.99 ( 0.60, 1.63) | 1.40 (0.74, 2.64) | 1.39 (0.73, 2.64) | 1.41 (0.74, 2.68) |  |
| ≥35 | 0.73 (0.41, 1.29) | 0.98 (0.51, 1.86) | 1.69 (0.72, 3.91) | 1.64 (0.69, 3.83) | 1.63 (0.69, 3.83) |  |
| **Paternal age (years), N** | 1673/152 | 1322/115 | 1322/115 | 1322/115 | 1322/115 |  |
| <25 | Ref | Ref | Ref | Ref | Ref |  |
| 25-34 | 0.76 (0.44, 1.30) | 0.66 (0.36, 1.21) | **0.46 (0.21, 0.97)*** | **0.44 (0.20, 0.94)*** | **0.43 (0.20, 0.93)*** |  |
| ≥35 | **0.54 (0.29, 0.99)*** | 0.51 (0.26, 1.00) | **0.31 (0.13, 0.74)**** | **0.29 (0.12, 0.71)**** | **0.29 (0.12, 0.71)**** |  |
| **Maternal education, N** | 1668/153 | 1322/115 | 1322/115 | 1322/115 | 1322/115 |  |
| Primary | ref | Ref | Ref | Ref | Ref |  |
| Secondary | 1.24 (0.67, 2.29) | 1.38 (0.62, 3.03) | 1.44 (0.63, 3.27) | 1.45 (0.64, 3.29) | 1.45 (0.64, 3.32) |  |
| Postsecondary | 1.27 (0.69, 2.33) | 1.47 (0.66, 3.20) | 1.54 (0.65, 3.61) | 1.52 (0.65, 3.57) | 1.53 (0.65, 3.60) |  |
| **Paternal education, N** | 1662/151 | 1322/115 | 1322/115 | 1322/115 | 1322/115 |  |
| Primary | Ref | Ref | Ref | Ref | Ref |  |
| Secondary | 0.82 (0.50, 1.32) | 0.96 (0.54, 1.70) | 0.88 (0.49, 1.57) | 0.88 (0.50, 1.58) | 0.88 (0.50, 1.58) |  |
| Postsecondary | 1.02 (0.62, 1.67) | 1.03 (0.57, 1.86) | 0.99 (0.53, 1.86) | 1.01 (0.53, 1.92) | 1.01 (0.53, 1.92) |  |
| **Parity, N** | 1683/153 | 1322/115 | 1322/115 | 1322/115 | 1322/115 |  |
| 1 | Ref | Ref | Ref | Ref | Ref |  |
| 2 | 0.88 (0.61, 1.26) | 0.73 (0.48, 1.11) | 0.76 (0.50, 1.18) | 0.80 (0.51, 1.24) | 0.83 (0.51, 1.26) |  |
| ≥3 | 0.97 (0.63, 1.48) | 0.86 (0.52, 1.42) | 0.92 (0.53, 1.61) | 0.98 (0.55, 1.73) | 0.98 (0.55, 1.73) |  |
| **Maternal BMI (kg/m^2^)^b^, N** | 1372/124 | 1322/115 | 1322/115 | 1322/115 | 1322/115 |  |
| <18.5 | *NA* | *NA* | *NA* | *NA* | *NA* |  |
| 18.5–24.9 | Ref | Ref | Ref | Ref | Ref |  |
| 25–29.9 | 1.31 (0.87, 1.98) | 1.31 (0.87, 1.99) | 1.39 (0.91, 2.12) | 1.41 (0.91, 2.17) | 1.41 (0.91, 2.17) |  |
| ≥30 | **2.31 (1.41, 3.77)***** | **2.10 (1.24, 3.54)***** | **2.27 (1.33, 3.87)**** | **2.26 (1.31, 3.87)**** | **2.26 (1.31, 3.88)**** |  |
| **Maternal smoking^b^, N** | 1626/141 | 1322/115 | 1322/115 | 1322/115 | 1322/115 |  |
| No | Ref | Ref | Ref | Ref | Ref |  |
| Yes | 0.84 (0.52, 1.35) | 0.82 (0.47, 1.43) | 0.85 (0.48, 1.51) | 0.86 (0.48, 1.53) | 0.86 (0.48, 1.53) |  |
| **Pregnancy characteristics** | | | | | | |
| **Assisted pregnancy IVF, N** | 1683/153 | 1322/115 | 1322/115 | 1322/115 | 1322/115 |  |
| No | Ref | Ref | Ref | Ref | Ref |  |
| Yes | 1.85 (0.76, 4.52) | 1.71 (0.63, 4.64) | 1.56 (0.56, 4.35) | 1.49 (0.53, 4.17) | 1.46 (0.52, 4.12) |  |
| **Mode of delivery, N** | 1683/153 | 1322/115 | 1322/115 | 1322/115 | 1322/115 |  |
| Vaginal no instruments | Ref | Ref | Ref | Ref | Ref |  |
| caesarean elective | 0.91 (0.40, 2.06) | 1.27 (0.56, 2.90) | 1.33 (0.58, 3.08) | 1.31 (0.56, 3.02) | 1.28 (0.55, 1.98) |  |
| caesarean emergency | 1.10 (0.61, 2.00) | 1.06 (0.51, 2.17) | 0.92 (0.43, 1.94) | 0.92 (0.42, 1.89) | 0.89 (0.42, 1.88) |  |
| forceps or vacuum | 1.25 (0.67, 2.31) | 1.44 (0.75, 2.78) | 1.38 (0.70, 2.72) | 1.37 (0.69, 2.70) | 1.36 (0.58, 2.68) |  |
| **Neonatal characteristics** | | | | | | |
| **GA (weeks), N** | 1683/153 | 1322/115 | 1322/115 | 1322/115 | 1322/115 |  |
| <37 | 0.99 (0.52, 1.89) | 1.20 (0.59, 2.48) | 1.24 (0.60, 2.57) | 1.24 (0.59, 2.60) | 1.24 (0.59, 2.60) |  |
| 37 – 41 | Ref | Ref | Ref | Ref | Ref |  |
| ≥42 | 1.14 (0.64, 2.01) | 1.13 (0.59, 2.17) | 1.03 (0.53, 2.00) | 1.05 (0.54, 2.03) | 1.05 (0.54, 2.03) |  |
| **Birthweight for GA**^c^**, N** | 1677/152 | 1318/114 | 1318/114 | 1318/114 | 1318/114 |  |
| AGA | Ref | Ref | Ref | Ref | Ref |  |
| SGA | - | - | - | - | - |  |
| LGA | 1.32 (0.69, 2.52) | 1.07 (0.47, 2.43) | 0.94 (0.41, 2.17) | 0.92 (0.39, 2.13) | 0.93 (0.40, 2.15) |  |
| **Child infection-I**^d^**, N** | 1606 /146 | 1257/112 | 1257/112 | 1257/112 | 1257/112 |  |
| No | Ref | Ref | Ref | Ref | Ref |  |
| Yes | *NA* | *NA* | *NA* | *NA* | *NA* |  |
| **5-min Apgar, N** | 1673/153 | 1315/115 | 1315/115 | 1315/115 | 1315/115 |  |
| ≥7 | Ref | Ref | Ref | Ref | Ref |  |
| <7 | *NA* | *NA* | *NA* | *NA* | *NA* |  |
| **Neonatal care^e^, N** | 1199/109 | 1063/95 | 1063/95 | 1063/95 | 1063/95 |  |
| No | Ref | Ref | Ref | Ref | Ref |  |
| Yes | 1.44 (0.83, 2.48) | 1.52 (0.85, 2.74) | 1.52 (0.84, 2.76) | 1.53 (0.84, 2.79) | 1.59 (0.81, 3.11) |  |

N, n of total observations/n of events, GA – gestational age; IVF – in vitro fertilisation; BMI – body mass index; AGA – adequate for GA, SGA- small for GA, LGA - large for GA; NA – less than 10 observations.

*** p < 0.001, ** p < 0.01, * p < 0.05; models 1 to 3 – shaded are perinatal characteristics used as adjustment covariates in the respective model.

^a^ – according to complete data for all used adjustment covariates; ^b^– smoking and BMI at the time of enrolment into maternal health care; ^c^– calculated according to birthweight, sex and gestational age; ^d^ – data according to the incoming patient registry; ^e^ – data available since 1995.
