# Supplementary material for: Parental, pregnancy and neonatal characteristics during the perinatal period as potential risk factors for childhood cancer: FeToxCancer case-control study
Source: PLoS One. 2026 Apr 16;21(4):e0333752. doi: 10.1371/journal.pone.0333752 (PMC13086354; doi:10.1371/journal.pone.0333752)
Supplement: S5 Table — (DOCX) [file pone.0333752.s005.docx]

S5 Table. Associations of perinatal characteristics with other cancer types combined (ICCC groups IV to XII, including by ICCC unclassified).

| **Perinatal characteristics** | Crude HR (95%CI) | Crude HR (95%CI)  Complete data^a^ | **Model 1**  HR (95%CI) | **Model 2**  HR (95%CI) | **Model 3**  HR (95%CI) |  |
| --- | --- | --- | --- | --- | --- | --- |
| **Parents characteristics** | | | | | | |
| **Maternal cancer, N** | 5687/517 | 4491/411 | 4491/411 | 4491/411 | 4491/411 |  |
| No | ref | Ref | Ref | Ref | Ref |  |
| Yes | 1.00 (0.79, 1.28) | 1.03 (0.78, 1.36) | 1.05 (0.80, 1.39) | 1.07 (0.81, 1.41) | 1.07 (0.82, 1.42) |  |
| **Maternal age (years), N** | 5687/517 | 4491/411 | 4491/411 | 4491/411 | 4491/411 |  |
| <25 | ref | Ref | Ref | Ref | Ref |  |
| 25-34 | 1.03 (0.82, 1.30) | 0.97 (0.74, 1.26) | 0.93 (0.68, 1.27) | 0.92 (0.66, 1.25) | 0.91 (0.66, 1.25) |  |
| ≥35 | *1.30 (0.97, 1.73)* | 1.16 (0.83, 1.62) | 0.98 (0.64, 1.50) | 0.94 (0.61, 1.44) | 0.93 (0.61, 1.44) |  |
| **Paternal age (years), N** | 5633 /511 | 4491/411 | 4491/411 | 4491/411 | 4491/411 |  |
| <25 | Ref | Ref | Ref | Ref | Ref |  |
| 25-34 | 0.94 (0.66, 1.34) | 0.87 (0.58, 1.29) | 0.89 (0.57, 1.38) | 0.88 (0.57, 1.37) | 0.91 (0.64, 1.41) |  |
| ≥35 | 1.17 (0.82, 1.68) | 1.11 (0.74, 1.65) | 1.11 (0.69, 1.80) | 1.10 (0.68, 1.79) | 1.13 (0.65, 1.85) |  |
| **Maternal education, N** | 5643/516 | 4491/411 | 4491/411 | 4491/411 | 4491/411 |  |
| Primary | ref | Ref | Ref | Ref | Ref |  |
| Secondary | 1.03 (0.75, 1.40) | 0.90 (0.64, 1.28) | 0.94 (0.66, 1.34) | 0.93 (0.65, 1.33) | 0.94 (0.66, 1.34) |  |
| Postsecondary | 1.13 (0.83, 1.54) | 0.94 (0.67, 1.32) | 0.95 (0.65, 1.39) | 0.95 (0.65, 1.38) | 0.96 (0.66, 1.40) |  |
| **Paternal education, N** | 5584/ 509 | 4491/411 | 4491/411 | 4491/411 | 4491/411 |  |
| Primary | Ref | Ref | Ref | Ref | Ref |  |
| Secondary | 0.87 (0.67, 1.12) | 0.85 (0.64, 1.13) | 0.86 (0.64, 1.15) | 0.85 (0.64, 1.14) | 0.85 (0.64, 1.14) |  |
| Postsecondary | 0.96 (0.74, 1.26) | 0.93 (0.69, 1.25) | 0.91 (0.65, 1.26) | 0.90 (0.65, 1.26) | 0.91 (0.65, 1.26) |  |
| **Parity, N** | 5687/517 | 4491/411 | 4491/411 | 4491/411 | 4491/411 |  |
| 1 | Ref | Ref | Ref | Ref | Ref |  |
| 2 | 1.06 (0.87, 1.29) | 1.06 (0.85, 1.31) | 1.03 (0.82, 1.30) | 1.05 (0.83, 1.32) | 1.05 (0.83, 1.33) |  |
| ≥3 | 1.09 (0.87, 1.37) | 1.10 (0.85, 1.42) | 1.03 (0.78, 1.37) | 1.05 (0.78, 1.41) | 1.05 (0.78, 1.41) |  |
| **Maternal BMI (kg/m^2^)^b^, N** | 4707 /426 | 4491/411 | 4491/411 | 4491/411 | 4491/411 |  |
| <18.5 | 0.98 (0.56, 1.71) | 1.03 (0.58, 1.79) | 1.03 (0.59, 1.30) | 1.02 (0.59, 1.80) | 1.02 (0.58, 1.78) |  |
| 18.5–24.9 | Ref | Ref | Ref | Ref | Ref |  |
| 25–29.9 | 1.00 (0.80, 1.26) | 1.03 (0.82, 1.30) | 1.03 (0.81, 1.30) | 1.01 (0.80, 1.28) | 1.01 (0.80, 1.28) |  |
| ≥30 | 1.02 (0.73, 1.41) | 1.04 (0.75, 1.45) | 1.01 (0.72, 1.41) | 0.97 (0.69, 1.36) | 0.97 (0.69, 1.36) |  |
| **Maternal smoking^b^, N** | 5467/491 | 4491/411 | 4491/411 | 4491/411 | 4491/411 |  |
| No | Ref | Ref | Ref | Ref | Ref |  |
| Yes | 0.86 (0.67, 1.10) | 0.87 (0.66, 1.16) | 0.85 (0.63, 1.14) | 0.84 (0.62, 1.13) | 0.83 (0.61, 1.12) |  |
| **Pregnancy characteristics** | | | | | | |
| **Assisted pregnancy IVF, N** | 5687/517 | 4491/411 | 4491/411 | 4491/411 | 4491/411 |  |
| No | Ref | Ref | Ref | Ref | Ref |  |
| Yes | 0.71 (0.36, 1.44) | 0.72 (0.34, 1.53) | 0.69 (0.33, 1.48) | 0.66 (0.31, 1.41) | 0.65 (0.31, 1.39) |  |
| **Mode of delivery, N** | 5687/517 | 4491/411 | 4491/411 | 4491/411 | 4491/411 |  |
| Vaginal no instruments | Ref | Ref | Ref | Ref | Ref |  |
| caesarean elective | **1.45 (1.03, 2.05)*** | **1.55 (1.07, 2.26)*** | **1.55 (1.06, 2.26)*** | **1.54 (1.07, 2.25)*** | **1.52 (1.04, 2.22)*** |  |
| caesarean emergency | **1.29 (1.01, 1.75)*** | **1.39 (1.00, 1.94)*** | **1.42 (1.01, 1.98)*** | **1.44 (1.03, 2.02)*** | 1.39 (0.98, 1.96) |  |
| forceps or vacuum | 0.98 (0.66, 1.48) | 1.06 (0.69, 1.63) | 1.11 (0.71, 1.72) | 1.11 (0.71, 1.73) | 1.11 (0.71, 1.82) |  |
| **Neonatal characteristics** | | | | | | |
| **GA (weeks), N** | 5687/517 | 4491/411 | 4491/411 | 4491/411 | 4491/411 |  |
| <37 | 1.22 (0.89, 1.69) | 1.36 (0.95, 1.94) | 1.36 (0.95, 1.95) | 1.26 (0.87, 1.82) | 1.26 (0.87, 1.82) |  |
| 37 – 41 | Ref | Ref | Ref | Ref | Ref |  |
| ≥42 | 0.91 (0.63, 1.32) | 0.99 (0.67, 1.49) | 0.99 (0.66, 1.48) | 0.98 (0.65, 1.46) | 0.98 (0.65, 1.46) |  |
| **Birthweight for GA**^c^**, N** | 5665/515 | 4476/410 | 4476/410 | 4476/410 | 4476/410 |  |
| AGA | Ref | Ref | Ref | Ref | Ref |  |
| SGA | 1.02 (0.67, 1.55) | 0.88 (0.51, 1.49) | 0.88 (0.51, 1.51) | 0.82 (0.48, 1.41) | 0.79 (0.45, 1.36) |  |
| LGA | 1.23 (0.83, 1.79) | 1.48 (0.96, 2.17) | 1.48 (0.95, 2.19) | 1.44 (0.97, 2.13) | 1.45 (0.98, 2.14) |  |
| **Child infection-I**^d^**, N** | 5423/493 | 4286/391 | 4286/391 | 4286/391 | 4286/391 |  |
| No | Ref | Ref | Ref | Ref | Ref |  |
| Yes | 0.79 (0.45, 1.37) | 0.95 (0.53, 1.70) | 0.95 (0.53, 1.68) | 0.93 (0.52, 1.66) | 0.93 (0.52, 1.64) |  |
| **5-min Apgar, N** | 5666/514 | 4481/409 | 4481/409 | 4481/409 | 4481/409 |  |
| ≥7 | Ref | Ref | Ref | Ref | Ref |  |
| <7 | **2.39 (1.38, 4.14)**** | **2.55 (1.36, 4.77)**** | **2.57 (1.36, 4.84)**** | **2.22 (1.17, 4.24)*** | **2.15 (1.12, 4.12)*** |  |
| **Neonatal care^e^, N** | 4103/373 | 3604/326 | 3604/326 | 3604/326 | 3604/326 |  |
| No | Ref | Ref | Ref | Ref | Ref |  |
| Yes | **1.49 (1.13, 2.00)**** | **1.48 (1.10. 1.99)**** | **1.50 (1.11, 2.02)**** | **1.38 (1.01, 1.89)*** | **1.40 (1.02, 1.97)*** |  |

N, n of total observations/n of events, GA – gestational age; IVF – in vitro fertilisation; BMI – body mass index; AGA – adequate for GA, SGA- small for GA, LGA - large for GA; NA – less than 10 observations.

*** p < 0.001, ** p < 0.01, * p < 0.05; models 1 to 3 – shaded are perinatal characteristics used as adjustment covariates in the respective model.

^a^ – according to complete data for all used adjustment covariates; ^b^– smoking and BMI at the time of enrolment into maternal health care; ^c^– calculated according to birthweight, sex and gestational age; ^d^ – data according to the incoming patient registry; ^e^ – data available since 1995.
