# Supplementary material for: Parental, pregnancy and neonatal characteristics during the perinatal period as potential risk factors for childhood cancer: FeToxCancer case-control study
Source: PLoS One. 2026 Apr 16;21(4):e0333752. doi: 10.1371/journal.pone.0333752 (PMC13086354; doi:10.1371/journal.pone.0333752)
Supplement: S6 Table — (DOCX) [file pone.0333752.s006.docx]

S6 Table. Associations of perinatal characteristics with risk of childhood cancer and cancer types after exclusion of children diagnosed with cancer-predisposing syndromes^#^.

| **Perinatal characteristics** | **Overall childhood cancer**  Crude HR (95%CI) | **Leukaemia**  HR (95%CI) | **CNS tumors**  HR (95%CI) | **Lymphoma**  HR (95%CI) | **Other cancer types combined**  HR (95%CI) |  |
| --- | --- | --- | --- | --- | --- | --- |
| **Parents characteristics** | | | | | | |
| **Maternal cancer, N** | 11778/1055 | 3108/280 | 2902/ 257 | 1319/115 | 4480/405 |  |
| No | ref | Ref | Ref | Ref | Ref |  |
| Yes | 1.02 (0.85, 1.21) | 1.04 (0.72, 1.50) | 0.89 (0.61, 1.30) | 1.09 (0.63, 1.89) | 1.08 (0.82, 1.43) |  |
| **Maternal age (years) , N** | 11778/1055 | 3108/280 | 2902/ 257 | 1319/115 | 4480/405 |  |
| <25 | ref | Ref | Ref | Ref | Ref |  |
| 25-34 | 1.11 (0.90, 1.35) | 1.09 (0.74, 1.60) | 1.43 (0.95, 2.16) | 1.41 (0.74, 2.68) | 0.92 (0.60, 1.25) |  |
| ≥35 | 1.07 (0.82. 1-39) | 1.12 (0.67, 1.86) | 1.14 (0.66, 1.99) | 1.63 (070, 3.84) | 0.93 (0.72, 1.44) |  |
| **Paternal age (years), N** | 11778/1055 | 3108/280 | 2902/ 257 | 1319/115 | 4480/405 |  |
| <25 | Ref | Ref | Ref | Ref | Ref |  |
| 25-34 | 1.11 (0.85, 1.47) | 0.76 (0.42, 1.34) | 1.18 (0.67, 2.09) | **0.43 (0.20, 0.93)*** | 1.13 (0.72, 1.75) |  |
| ≥35 | 1.11 (0.96, 1-28) | 1.08 (0.82, 1.42) | 1.15 (0.85, 1.53) | **0.29 (0.12, 0.71)**** | 1.28 (0.89, 11.62) |  |
| **Maternal education, N** | 11778/1055 | 3108/280 | 2902/ 257 | 1319/115 | 4480/405 |  |
| Primary | ref | Ref | Ref | Ref | Ref |  |
| Secondary | 0.95 (0.76, 1.19) | 0.93 (0.61, 1.43) | 0.86 (0.54, 1.35) | 1.46 (0.64, 3.32) | 0.93 (0.65, 1.33) |  |
| Postsecondary | 0.96 (0.76, 1.22) | 0.87 (0.55, 1.36) | 0.87 (0.54, 1.41) | 1.54 (0.65, 3.62) | 0.94 (0.64, 1.37) |  |
| **Paternal education, N** | 11778/1055 | 3108/280 | 2902/ 257 | 1319/115 | 4480/405 |  |
| Primary | Ref | Ref | Ref | Ref | Ref |  |
| Secondary | 1.01 (0.84, 1.22) | 1.15 (0.79, 1.52) | 1.27 (0.83, 1.92) | 0.88 (0.50, 1.59) | 0.85 (0.63, 1.14) |  |
| Postsecondary | 1.02 (0.82, 1.26) | 0.96 (0.63, 1.42) | 1.37 (0.87, 2.16) | 1.02 (0.54, 1.93) | 0.90 (0.64, 1.24) |  |
| **Parity, N** | 11778/1055 | 3108/280 | 2902/ 257 | 1319/115 | 4480/405 |  |
| 1 | Ref | Ref | Ref | Ref | Ref |  |
| 2 | 1.02 (0.88, 1.18) | 1.15 (0.87, 1.52) | 0.91 (0.69, 1.22) | 0.80 (0.52, 1.25) | 1.07 (0.83, 1.35) |  |
| ≥3 | 0.98 (0.81, 1.18) | 1.14 (0.80, 1.63) | 0.69 (0.47, 1.03) | 0.98 (0.56, 1.74) | 1.06 (0.78, 1.42) |  |
| **Maternal BMI (kg/m^2^)^b^, N** | 11778/1055 | 3108/280 | 2902/ 257 | 1319/115 | 4480/405 |  |
| <18.5 | 1.21 (0.85, 1.75) | **2.51 (1.45, 4.36)**** | 1.00 (0.41, 2.45) | *NA* | 0.97 (0.54, 1.73) |  |
| 18.5–24.9 | Ref | Ref | Ref | Ref | Ref |  |
| 25–29.9 | 1.15 (0.99, 1.33) | 1.22 (0.93, 1.61) | 1.18 (0.88, 1.58) | 1.41 (0.91, 2.17) | 1.03 (0.81, 1.30) |  |
| ≥30 | 1.16 (0.95, 1.42) | 0.89 (0.59, 1.35) | **1.41 (1.02, 2.08)*** | **2.24 (1.31, 3.86)**** | 0.97 (0.69, 1.36) |  |
| **Maternal smoking^b^, N** | 11778/1055 | 3108/280 | 2902/ 257 | 1319/115 | 4480/405 |  |
| No | Ref | Ref | Ref | Ref | Ref |  |
| Yes | 0.92 (0.75, 1.10) | 0.99 (0.68, 1.42) | 0.98 (0.66, 1.45) | 0.86 (0.48, 1.53) | 0.84 (0.63, 1.14) |  |
| **Pregnancy characteristics** | | | | | | |
| **Assisted pregnancy IVF, N** | 11778/1055 | 3108/280 | 2902/ 257 | 1319/115 | 4480/405 |  |
| No | Ref | Ref | Ref | Ref | Ref |  |
| Yes | 0.88 (0.57, 1.36) | 1.13 (0.52, 2.46) | 0.73 (0.27, 2.00) | 1.46 (0.52, 4.12) | 0.67 (0.31, 1.44) |  |
| **Mode of delivery, N** | 11778/1055 | 3108/280 | 2902/ 257 | 1319/115 | 4480/405 |  |
| Vaginal no instruments | Ref | Ref | Ref | Ref | Ref |  |
| caesarean elective | 1.05 (0.80, 1.37) | 0.83 (0.47, 1.48) | 0.74 (0.39, 1.40) | 1.28 (0.54, 2.96) | **1.44 (1.03, 2.05)*** |  |
| caesarean emergency | 1.18 (0.94, 1.47) | 1.28 (0.83, 1.98) | 0.97 (0.62, 1.52) | 0.90 (0.42, 1.91) | 1.35 (0.95, 1.92) |  |
| forceps or vacuum | 1.11 (0.85, 1.44) | 1.08 (0.64, 1.82) | 0.99 (0.58, 1.67) | 1.39 (0.59, 2.74) | 1.11 (0.71, 1.82) |  |
| **Neonatal characteristics** | | | | | | |
| **GA (weeks), N** |  | 3108/280 | 2902/ 257 | 1319/115 | 4480/405 |  |
| <37 | 1.07 (0.83, 1.38) | 0.76 (0.42, 1.39) | 1.15 (0.70, 1.88) | 1.24 (0.59, 2.60) | 1.16 (0.79, 1.71) |  |
| 37 – 41 | Ref | Ref | Ref | Ref | Ref |  |
| ≥42 | 0.98 (0.77, 1.26) | 0.95 (0.58, 1.56) | 1.00 (0.60, 1.67) | 1.05 (0.54, 2.03) | 0.98 (0.66, 1.47) |  |
| **Birthweight for GA**^c^**, N** | 11742/1042 | 3098/279 | 2895/254 | 1315/114 | 4465/404 |  |
| AGA | Ref | Ref | Ref | Ref | Ref |  |
| SGA | 0.90 (0.65, 1.25) | 0.79 (0.43, 1.44) | 0.99 (0.50, 1.95) | - | 0.76 (0.43, 1.33) |  |
| LGA | **1.22 (1.01, 1.59)*** | **1.59 (1.01, 2.57)*** | 0.97 (0.52, 1.81) | 0.92 (0.40, 2.14) | 1.30 (0.861.96) |  |
| **Child infection-I**^d^**, N** | 11325/1016 | 3037/274 | 2789/247 | 1254/112 | 4276/385 |  |
| No | Ref | Ref | Ref | Ref | Ref |  |
| Yes | 1.05 (0.74, 1.48) | 1.32 (0.76, 2.50) | *NA* | *NA* | 0.94 (0.54, 1.68) |  |
| **5-min Apgar, N** | 11735/1048 | 3097/278 | 2887/254 | 1312/115 | 4470/403 |  |
| ≥7 | Ref | Ref | Ref | Ref | Ref |  |
| <7 | 1.39 (0.89, 2.37) | *NA* | *NA* | *NA* | **1.81 (1.02, 3.74)*** |  |
| **Neonatal care^e^, N** | 11154/1014 | 2669/240 | 2508/221 | 1061/95 | 3595/320 |  |
| No | Ref | Ref | Ref | Ref | Ref |  |
| Yes | 1.14 (0.90, 1.45) | 1.03 (0.62, 1.68) | 0.85 (0.51, 1.43) | 1.62 (0.83, 3.20) | 1.32 (0.93, 1.88) |  |

N, n of total observations/n of events, GA – gestational age; IVF – in vitro fertilisation; BMI – body mass index; AGA – adequate for GA, SGA- small for GA, LGA - large for GA;

NA – less than 10 observations.

*** p < 0.001, ** p < 0.01, * p < 0.05; shaded are perinatal characteristics used as adjustment covariates in the respective model.

^#^ including: Down syndrome, ICD9: 758A and ICD10:Q90; Neurofibromatosis type 1, ICD9: 237H and ICD10: Q850; Congenital malformation syndromes involving early overgrowth, ICD9: 756W, 259W and ICD10: Q873; and Von Hippel-Lindau syndrome, ICD9: 759G and ICD10: Q858)

^a^ according to complete data for all used adjustment covariates.

^b^smoking and BMI at the time of enrolment into maternal health care.

^c^calculated according to birthweight, sex and gestational age.

^d,^ data according to the incoming patient registry.

^e^data available since 1995.
