# Supplementary material for: Parental, pregnancy and neonatal characteristics during the perinatal period as potential risk factors for childhood cancer: FeToxCancer case-control study
Source: PLoS One. 2026 Apr 16;21(4):e0333752. doi: 10.1371/journal.pone.0333752 (PMC13086354; doi:10.1371/journal.pone.0333752)
Supplement: S7 Table — (DOCX) [file pone.0333752.s007.docx]

S7 Table. Association of birthweight for GA with risk of overall childhood cancer and leukaemia after additional adjustment for gestational diabetes.

| **Birthweight for GA** | **Overall childhood cancer**  N | **Leukaemia**  N |
| --- | --- | --- |
|  | adjusted HR^a^ (95%CI) | adjusted HR^a^ (95%CI) |
|  | 8396/769 | 2406/225 |
| AGA | Ref | Ref |
| SGA | 0.96 (0.66, 1.40) | - |
| LGA | **1.32 (1.00, 1.77)*** | 1.57 (0.96, 2.63) |

N, n of total observations/n of events;* p <0.05,^a^- adjusted according to model 3 and additionally for Gestational diabetes
