# Supplementary material for: Parental, pregnancy and neonatal characteristics during the perinatal period as potential risk factors for childhood cancer: FeToxCancer case-control study
Source: PLoS One. 2026 Apr 16;21(4):e0333752. doi: 10.1371/journal.pone.0333752 (PMC13086354; doi:10.1371/journal.pone.0333752)
Supplement: S8 Table — (DOCX) [file pone.0333752.s008.docx]

S8 Table. Association of 5-min Apgar with other cancer types combined after additional adjustment for birthweight for GA and neonatal care (a) and distribution of 5-min Apgar according to the age at diagnosis (b).

*a)*

| **5-min Apgar score** | **Other cancer types combined**  N  adj HR^a^ (95%CI) |
| --- | --- |
| 7-10 | 3582/324  Ref |
| 0-6 | **2.10 (1.04, 4.27) *** |

N, n of total observations/n of events; * p <0.05.

^a^- adjusted according to model 3, and additionally for birthweight for GA and neonatal care

*b)*

| **Age at**  **diagnosis** | **5-min**  **Apgar score** | **Overall childhood cancer** | |
| --- | --- | --- | --- |
|  |  | Cases, N (%) | Controls, N (%) |
| ≤6 months | 7-10 | 99 (94) | 1047 (99) |
|  | 0-6 | 6 (6) | 9 (1) |
| ≤1year | 7-10 | 253 (97) | 2598 (99) |
|  | 0-6 | 9 (3) | 27 (1) |
| >1 year | 7-10 | 1208 (98.5) | 10597(98) |
|  | 0-6 | 16 (1.5) | 122 (1) |
