# Supplementary material for: Parental, pregnancy and neonatal characteristics during the perinatal period as potential risk factors for childhood cancer: FeToxCancer case-control study
Source: PLoS One. 2026 Apr 16;21(4):e0333752. doi: 10.1371/journal.pone.0333752 (PMC13086354; doi:10.1371/journal.pone.0333752)
Supplement: S9 Table — (DOCX) [file pone.0333752.s009.docx]

S9 Table. Distribution and association of specific neonatal therapies with overall childhood cancer (a), and association of mechanical ventilation with other cancer types (b).

a)

|  | **Overall childhood cancer** | | | |  |
| --- | --- | --- | --- | --- | --- |
| **Neonatal treatment** | Case N(%) | Control N(%) | N  adj HR^a^(95%CI) | N  adj HR^b^(95%CI) | N  adj HR^c^(95%CI) |
| **Mechanical ventilation** |  |  | 9832/895 | 9766/885 | 9802/874 |
| No | 954 (94) | 9805 (97) | Ref | Ref | Ref |
| Yes | 60 (6) | 335 (3) | **1.88 (1.39, 2.53)***** | **1.79 (1.31, 2.43)***** | **1.51 (1.07, 2.16)** |
| **Phototherapy^d^** |  |  |  |  |  |
| No | 627 (99) | 6296 (99) | Ref | Ref | Ref |
| Yes | 8 (1.3) | 54 (0.8) | NA | NA | NA |
| **Steroid treatment** |  |  |  |  |  |
| No | 1013 (99.9) | 10112 (99.8) | Ref | Ref | Ref |
| Yes | 1 (0.1) | 18 (0.2) | NA | NA | NA |
| **Antibiotic therapy^d^** |  |  |  |  |  |
| No | 626 (98.6) | 6297 (99.2) | Ref | Ref | Ref |
| Yes | 9 (1.4) | 53 (0.8) | NA | NA | NA |
| **Surfactant tretament^d^** |  |  |  |  |  |
| No | 634 (99.8) | 6337 (99.8) | Ref | Ref | Ref |
| Yes | 1 (0.2) | 13 (0.2) | NA | NA | NA |

N, n of total observations/n of events; *** p < 0.001.

^a^, adjusted according to model 3,

^b^, adjusted according to model 3 and additionally for 5-min Apgar score and birthweight for GA.

^c^, adjusted according to model 3 with exclusion of children with diagnosed cancer predisposing syndrome.

^d^,data available only from SNQ Register (2002-2021);

b)

| **Mechanical**  **ventilation** |  | | |  |  |
| --- | --- | --- | --- | --- | --- |
|  | Case N(%) | Control N(%) | N  adj HR^a^(95%CI) | N  adj HR^b^(95%CI) | N  adj HR^c^(95%CI) |
| **Leukemia** | | | | |  |
| No | 260 (95) | 2656 (97) | 2681/249  Ref | 2662/247  Ref | 2669/240  Ref |
| Yes | 14 (5) | 84 (3) | 1.24 (0.61, 2.53) | 1.22 (0.58, 2.59) | 0.97 (0.43, 2.15) |
| **CNS tumor** | | | | |  |
| No | 243 (93) | 2506 (96) | 2516/228  Ref | 2498/223  Ref | 2508/221  Ref |
| Yes | 18 (7) | 104 (4) | 1.44 (0.75, 2.77) | 1.54 (0.79, 2.99) | 1.44 (0.73, 2.83) |
| **Lymphoma** | | | | |  |
| No | 104 (95) | 1058 (97) | 1063/95  Ref | 1057/94  Ref | 1061/95  Ref |
| Yes | 5 (5) | 32 (3) | **-** | **-** | **-** |
| **Other cancer types combined** | | | | |  |
| No | 350 (94) | 3613 (97) | 3604/326  Ref | 3582/324  Ref | 3595/320  Ref |
| Yes | 23 (6) | 117 (3) | **2.15 (1.29, 3.56)**** | **1.86 (1.08, 3.21)*** | **1.84 (1.12, 3.32)*** |

N, n of total observations/n of events; ** p < 0.01, * p < 0.05.

^a^, adjusted according to model 3,

^b^, adjusted according to model 3 and additionally for 5-min Apgar score and birthweight for GA,

^c^, adjusted according to model 3 with exclusion of children with diagnosed cancer predisposing syndrome
