# Supplementary material for: Parental, pregnancy and neonatal characteristics during the perinatal period as potential risk factors for childhood cancer: FeToxCancer case-control study
Source: PLoS One. 2026 Apr 16;21(4):e0333752. doi: 10.1371/journal.pone.0333752 (PMC13086354; doi:10.1371/journal.pone.0333752)
Supplement: S10 Table — (DOCX) [file pone.0333752.s010.docx]

S10 Table. Association of maternal BMI with leukaemia, CNS tumour and lymphoma after additional adjustment for birthweight for GA (a) or gestational diabetes (b).

| **BMI (kg/m^2^)** | **Leukaemia** | **CNS tumour** | **Lymphoma** |
| --- | --- | --- | --- |
|  | N  Adj HR (95%CI) | N  Adj HR (95%CI) | N  Adj HR (95%CI) |
| **a** | 3114/290 | 2904/261 | 1318/114 |
| <18.5 | **2.48 (1.43, 4.32)**** | 1.00 (0.41, 2.47) | - |
| 18.5–24.9 | Ref | Ref | Ref |
| 25–29.9 | 1.18 (0.90, 1.55) | 1.19 (0.89, 1.59) | 1.41 (0.91, 2.18) |
| ≥30 | 0.89 (0.59, 1.33) | **1.52 (1.04, 2.23)*** | **2.29 (0.33, 3.96)**** |
| **b** | 2414/226 | 2138/195 | 869/80 |
| <18.5 | **2.30 (1.16, 4.57)*** | - | - |
| 18.5–24.9 | Ref | Ref | Ref |
| 25–29.9 | 1.22 (0.90, 1.66) | 1.26 (0.90, 1.75) | 1.42(0.85, 2.36) |
| ≥30 | 0.98 (0.63, 1.50) | **1.62 (1.07, 12.46)*** | **2.03 (1.06, 3.88)*** |

N, n of total observations/n of events; ** p < 0.01, * p < 0.05

a – adjusted according to model 3 and additionally for birthweight for GA

b – adjusted according to model 3 and additionally for gestational diabetes
