# Supplementary material for: Parental, pregnancy and neonatal characteristics during the perinatal period as potential risk factors for childhood cancer: FeToxCancer case-control study
Source: PLoS One. 2026 Apr 16;21(4):e0333752. doi: 10.1371/journal.pone.0333752 (PMC13086354; doi:10.1371/journal.pone.0333752)
Supplement: S11 Table — (DOCX) [file pone.0333752.s011.docx]

S11 Table. Association between mode of delivery and risk of other cancer types combined after additional adjustment for birthweight for GA and 5-min Apgar score.

| **Mode of delivery** | **Other cancer types combined** |
| --- | --- |
|  | N  Adj^a^ HR (95%CI) |
| Vaginal | 4466/408  Ref |
| Caesarean planned | **1.50 (1.02, 2.19)*** |
| Caesarean emergency | 1.34 (0.94, 1.99) |
| Forceps or vacuum | 1.07 (0.69, 1.68) |

N, n of total observations/n of events; *, p < 0.05

^a^, adjusted according to model 3 and additionally for birthweight for GA and 5-min Apgar

*Table S12: Association between parental 5-year advance in age with risk of childhood cancer and specific cancer types*

| **Parental age**  **per 5-years** | **Overall childhood cancer** | **Leukemia** | **CNS tumor** | **Lymphoma** | **Other cancer types combined** |
| --- | --- | --- | --- | --- | --- |
|  | N  Adj^a^ HR (95%CI) | N  Adj^a^ HR (95%CI) | N  Adj^a^ HR (95%CI) | N  Adj^a^ HR (95%CI) | N  Adj^a^ HR (95%CI) |
|  | 11816/1078 | 3124/291 | 1911/264 | 1322/115 | 4491/411 |
| Maternal Age | 1.00 (0.86, 1.17) | 1.00 (0.88, 1.15) | 1.09 (0.95, 1.27) | 0.90 (0.72, 1.12) | 1.01 (0.90, 1.14) |
| Paternal Age | 1.00 (0.94, 1.06) | 1.02 (0.88, 1.15) | 0.94 (0.83, 1.05) | 0.93 (0.83, 1.08) | 1.06 (0.97, 1.16) |

N*, n of total observations/n of events; ^a^, adjusted according to model 3.
