# Supplementary material for: Parental, pregnancy and neonatal characteristics during the perinatal period as potential risk factors for childhood cancer: FeToxCancer case-control study
Source: PLoS One. 2026 Apr 16;21(4):e0333752. doi: 10.1371/journal.pone.0333752 (PMC13086354; doi:10.1371/journal.pone.0333752)
Supplement: S13 Table — (DOCX) [file pone.0333752.s013.docx]

S13 Table. Distribution and associations of additionally tested perinatal characteristics with childhood cancer.

| **Perinatal characteristics** | **Overall childhood cancer** | | |
| --- | --- | --- | --- |
|  | Cases N(%^a^) | Controls  N(%^a^) | Crude HR (95%CI)  (No = Ref) |
| **Maternal smoking in 30-32 gest. week** |  |  |  |
| No/Yes | 633 (88) /85 (12) | 696 (89)/817 (11) | 0.96 (0.77, 1.21) |
| missing | 622 (46) | 5887 (44) |  |
| **Maternal diabetes^a^** |  |  |  |
| No/Yes | 846 (98)/19 (2) | 8418 (97)/233 (3) | 0.88 (0.56, 1.39) |
| Missing | 475 (35) | 4749 (35) |  |
| **Maternal Infection during pregnancy^b^** |  |  |  |
| No/Yes | 1283 (99.5)/6 (0.5) | 12804 (99.3)/86 (0.7) | 0.76 (0.34, 1.69) |
| missing | 51(4) | 510 (4) |  |
| **Child cancer predisposing syndrome (any)** |  |  |  |
| No /Yes | 1308 (97.6) /32 (2.4) | 13382 (99.9) / 18(0.13) | 10.9 (7.79, 15.5)*** |
| **Down syndrome** |  |  |  |
| No/Yes | 1325 (98.8) / 15 (1.1) | 12287 (99.9) / 13 (0.10) | 8.02 (4.82, 13.4)*** |
| **Neurofibromatosis type 1** |  |  |  |
| No/Yes | 1328 (99.1) / 12 (0.9) | 13398 (99.9) / 2 (0.00) | - |
| **Congenital malformation syndromes involving early overgrowth** |  |  |  |
| No/Yes | 1335 (99.6) / 5 (0.4) | 13399 (1000) / 1 (0.01) | - |
| **Von-Hippel Lindau syndrome** |  |  |  |
| No/Yes | 1340 (100)/- | 13397 (99.9) / 3 (0.02) | - |
| **Mother's country of birth** |  |  |  |
| Sweden | 1051 (78) | 10480 (78) | Ref |
| Nordic countries | 27 (2) | 293 (2) | 0.99 (0.68, 1.45) |
| Europe | 135 (10) | 1143 (8) | 1.17 (0.89, 1.51) |
| Afrika | 14 (1) | 237 (2) | 0.72 (0.42, 1.21) |
| Asia | 96 (7) | 1092 (8) | 1.02 (0.82, 1.26) |
| North America | 5 (0.4) | 39 (0.3) | - |
| South America | 10 (0.8) | 99 (0.7) | 1.06 (0.57, 1.99) |
| Oceania | 2 (0.2) | 6 (0.04) | - |
| Soviet Union | - | 10 (0.07) | - |
| missing | - | 1(0.01) |  |
| **Father's country of birth** |  |  |  |
| Sweden | 1048 (79) | 10222 (77) | Ref |
| Nordic countries | 30 (2) | 339 (3) | 0.92 (0.65, 1.33) |
| Europe | 123 (9) | 1227 (9) | 1.06 (0.88, 1.28) |
| Afrika | 16 (1) | 261 (2) | 0.70 (0.43, 1.15) |
| Asia | 96 (7) | 1067 (8) | 1.00 (0.82, 1.24) |
| North America | 6 (0.5) | 66 (0.5) | - |
| South America | 8 (0.6) | 93 (0.7) | - |
| Oceania | - | 8 (0.06) | - |
| Soviet Union | - | 5 (0.04) | - |
| missing | 13 (1) | 112 (1) |  |
| **Parent background^c^** |  |  |  |
| Swedish ancestry | 769 (58) | 7581 (57) | Ref |
| Foreign ancestry | 241 (18) | 2416 (18) | 1.13 (0.98, 1.31) |
| Mixed ancestry | 318 (24) | 3291 (25) | 0.98 (0.86, 1.11) |

^a^ – data available since 1997; ^b^ – based on the patient registry on in-patient care, ^c^- Swedish ancestry: both mother and father were born to Swedish parents; Foreign ancestry: both mother and father were born to non-Swedish parents, Mixed ancestry: one of the parents born to Swedish parents and one to foreign parents.
