# Supplementary material for: Parental, pregnancy and neonatal characteristics during the perinatal period as potential risk factors for childhood cancer: FeToxCancer case-control study
Source: PLoS One. 2026 Apr 16;21(4):e0333752. doi: 10.1371/journal.pone.0333752 (PMC13086354; doi:10.1371/journal.pone.0333752)
Supplement: S14 Table — (DOCX) [file pone.0333752.s014.docx]

S14 Table. Associations of birthweight for GA with maternal BMI and Gestational diabetes (a) and of 5-min Apgar with birthweight for GA, gestational age and admission to neonatal care (b).

*a)*

|  | **Birthweight for GA, N (%)** | | |
| --- | --- | --- | --- |
|  | **AGA** | **SGA** | **LGA** |
| Maternal BMI (kg/m^2^) |  |  |  |
| <18.5 | 292 (3) | 28 (6) | 6 (1) |
| 18.5–24.9 | 7140 (64) | 296 (62) | 238 (42) |
| 25–29.9 | 2695 (24) | 110 (23) | 192 (34) |
| ≥30 | 1124 (10) | 47 (10) | 137 (24) |
| Gestational diabetes |  |  |  |
| No | 8443 (98) | 356 (98) | 429 (94) |
| Yes | 214 (2) | 8 (2) | 29 (6) |

GA – gestational age; AGA-adequate for GA, SGA- small for GA, LGA – large for GA; BMI – body mass index

*b)*

|  | **5-min Apgar, N (%)** | |
| --- | --- | --- |
|  | **<7** | **≥7** |
| Birthweight for GA |  |  |
| AGA | 135 (79) | 13241 (92) |
| SGA | 25 (15) | 568 (4) |
| LGA | 10 (6) | 654 (5) |
| GA (weeks) |  |  |
| <37 | 61 (35) | 859 (6) |
| 37 – 41 | 98 (56) | 12697 (88) |
| ≥42 | 15 (9) | 946 (7) |
| Neonatal care |  |  |
| No | 40 (28) | 9883(90) |
| Yes | 105 (72) | 1087 (10) |

GA – gestational age; AGA-adequate for GA, SGA- small for GA, LGA – large for GA
