# Supplementary material for: Parental, pregnancy and neonatal characteristics during the perinatal period as potential risk factors for childhood cancer: FeToxCancer case-control study
Source: PLoS One. 2026 Apr 16;21(4):e0333752. doi: 10.1371/journal.pone.0333752 (PMC13086354; doi:10.1371/journal.pone.0333752)
Supplement: S1 Fig — (DOCX) [file pone.0333752.s015.docx]

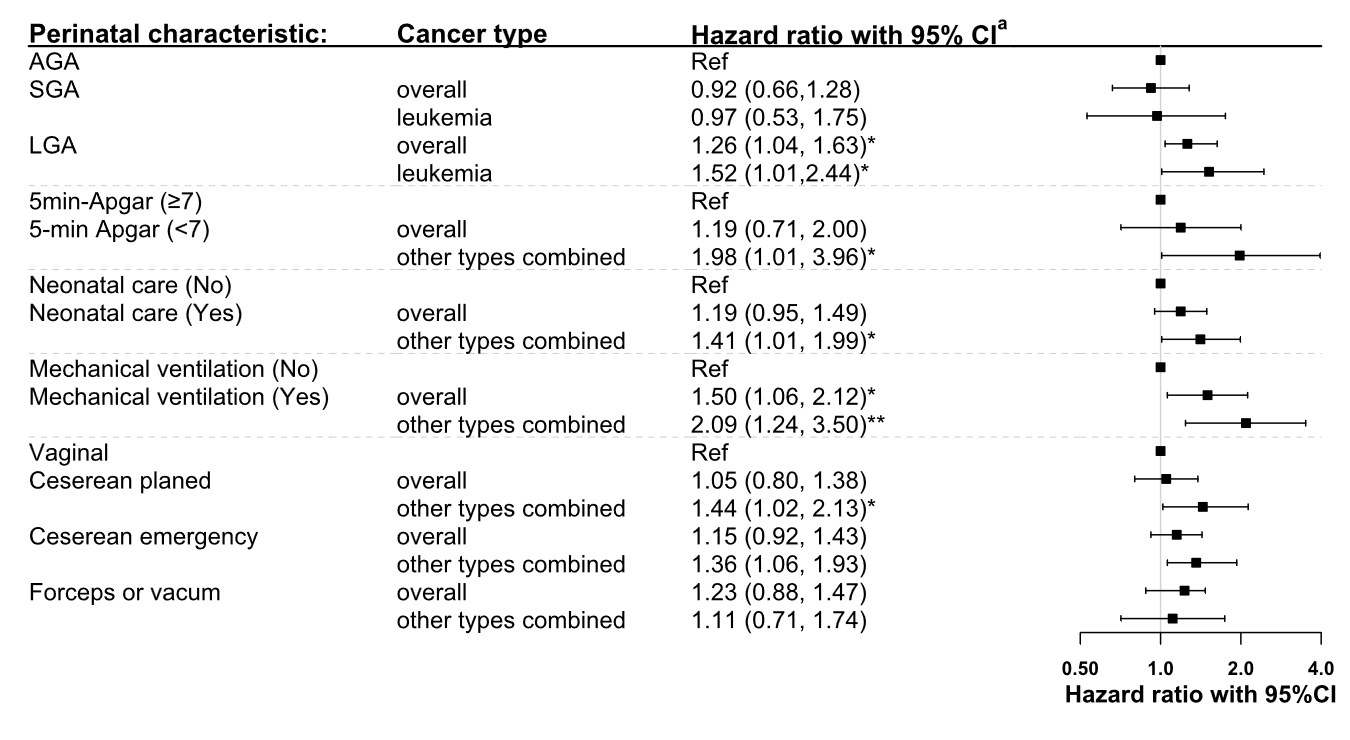


S1 Fig: Forest plot presenting associations of birthweight for GA, 5-min Apgar score, admission to neonatal care, mechanical ventilation treatment, and mode of delivery with the risk of overall childhood cancer, leukaemia or other cancer types combined after the exclusion of cancer cases diagnosed at birth (adjusted according to model 3; ** p < 0.01, *p < 0.05; other types combined– ICCC groups IV-XII including unclassified diagnoses; AGA – adequate for GA, SGA–small for GA, LGA–large for GA).
