# Supplementary material for: Parental, pregnancy and neonatal characteristics during the perinatal period as potential risk factors for childhood cancer: FeToxCancer case-control study
Source: PLoS One. 2026 Apr 16;21(4):e0333752. doi: 10.1371/journal.pone.0333752 (PMC13086354; doi:10.1371/journal.pone.0333752)
Supplement: Attachment — Submitted filename: Response to reviewers.docx [file pone.0333752.s017.docx]

Division of Occupational and Environmental Medicine

Lund University

Sweden

**Letter of response**

We would like to thank the editor and the reviewers for their time reviewing the manuscript and for their constructive comments and suggestions, which have improved the quality of the manuscript. We have addressed all comments and revised the manuscript accordingly. Our point-by-point responses are given below.

**Reviewer #1:**

The manuscript presents a large-scale population-based case-control study investigating associations between various perinatal factors and childhood cancer risk. The topic is timely, highly relevant, and aligns with PLOS ONE’s focus on scientifically rigorous, ethically conducted research that contributes meaningfully to its field.
Strengths:The study is based on a well-powered sample (1340 cases, 13,400 controls). Comprehensive use of Swedish national registries ensures high data reliability. The use of Cox regression with age as the underlying time scale is a strength over simple logistic regression. Results are adjusted in a stepwise fashion, providing robustness.

**Weaknesses / Suggestions:
-Collider bias is acknowledged, and the strategy of multiple models (with/without GA) is commendable. Still, a more detailed justification for the modelling strategy should be included in the Discussion.**

As suggested, we have included the following sentence in the discussion of limitations of the study: *“Preterm birth reflects both immaturity and various pathological factors, with currently unclear links to GA. Consequently, in studies on neonatal outcomes, adjusting for GA, as a potential mediator factor, may introduce collider bias (27). In our study, we examined numerous maternal and pregnancy characteristics, including GA, as potential risk factors for childhood cancer, many of which have unclear relationships with GA. To assess GA as both a potential risk factor and a true confounder (e.g. neonatal care, mechanical ventilation therapy) while addressing collider bias, we fitted models both with and without GA, as previously proposed by Wilcox et al., 2011 (27).”*

**-Multiple comparisons: The manuscript reports a large number of statistical tests. While correction was intentionally not applied, this choice should be more clearly defended in the limitations section.**

According to comments and suggestions of reviewers 1 and 2, the following was added to the discussion of limitations of the study: *“The statistical analysis includes multiple comparisons, increasing the risk of false positives; however, given the limited statistical power, lowering the significance threshold could potentially overlook biologically meaningful associations. Therefore, we chose to interpret the results cautiously in the context of existing literature rather than adjust for multiple comparisons, still acknowledging that some statistically significant associations may be due to chance. “*

-**Covariate selection: Certain known risk factors (e.g., environmental exposures, family history) are not accounted for. This should be addressed as a limitation.**

This information is included in the conclusion: *“Moreover, missing data for some characteristics (e.g., BMI), as well as lack of information on risk factors such as family history and environmental exposures, might represent bias in the current study.”*

**-IVF data: The availability from 1989-2006 and 2007-2021 in separate registries should be more clearly discussed in terms of its impact on data completeness.**

In this study, we have investigated whether assisted pregnancy with IVF (yes vs. no) was associated with the risk of childhood cancer. The Swedish National Quality Register of Assisted Reproduction (Q-IVF) was initiated in 2007; however, for the study period preceding the year of 2007, we could access the same information from the National Medical Birth Register for all study participants. As such, the inclusion of two separate registries to obtain this information did not impact the completeness of our data (i.e. information is available for the whole study population; see Table 2).

**Clarity of Writing
Strengths:The manuscript is overall clearly structured and easy to follow. The English is fluent and mostly well-polished.
Suggestions:
-Some paragraphs in the Discussion are overly long and could be made more concise.**

As suggested, we have made several discussion paragraphs more concise.

**-Avoid repetition when reporting results, especially when hazard ratios are reiterated in both Results and Discussion.**

We have removed the repetitions of the results within the discussion.
**-Minor language edits are needed: Consider proofreading by a native speaker or editor for minor grammatical refinements.**
As suggested, the manuscript was proofread by a native speaker.

**Reviewer #2:** In this manuscript the authors have examined the association between several perinatal characteristics and the occurrence of childhood cancer using a register-based case-control data that includes all children diagnosed with cancer in southern Sweden between 1989-2021. The authors found several statistically significant associations: while these associations have been reported in previous studies, results were often conflicting. My main comment to the authors is that I believe that they should have used a conditional logistic regression model to analyze their data instead of a Cox proportional hazards model. See below for more details on this as well as additional comments on the manuscript.

**- Abstract: please indicate that the numbers in parentheses represent hazard ratios and 95% confidence intervals.**

As suggested, we have added HR, 95CI% into all parentheses.

- **Abstract: add to the abstract that the 1340 cancer cases were born** **in southern Sweden between 1989-2021**

Revised as suggested.

- **Regarding controls selection, the authors wrote “The inclusion criteria were being born between 1989 and 2021 with no prior history of cancer diagnosis or recorded death before the age of 19, while allowing for cancer diagnosis at the age of 19 or older.” I guess that also controls did not emigrate before age 19, or otherwise you wouldn’t know whether they died or got a cancer diagnosis. However, since the controls cannot die before age 19 and some of the assessed perinatal characteristics are linked to childhood mortality (especially infant mortality) I wonder whether the decision to select controls only among children who didn’t die before age 19 could have led to a slight overestimation of the risk estimates, as these controls likely had more normal perinatal characteristics than the general population (that includes children who died during childhood). Moreover, I think that this sentence should be rephrased since it is not possible to check whether potential controls born in the more recent years are cancer-free at age 19 (or have not died by age 19) since they have not reached this age during the study period**

According to the reviewer`s comment this part was rephrased as follows: “*The inclusion criteria were being born between 1989 and 2021 with no known cancer diagnosis or recorded death before the age of 19, emigration or end of the study, while allowing for cancer diagnosis at the age of 19 or older. “*

- **Small for gestational age is defined as a birth weight below the 10th percentile for a specific gestational age, and these percentiles are usually estimated separately for boys and girls. This means that, in the general population, there are approximately 10% of children that are born SGA. However, when defining SGA as SD < -2, there will be a bit more than 2-3% of children defined as being born SGA (in Table 1 the authors reported that it was 4%, which is much lower than the definition of SGA). The same reasoning applies to LGA. Can the authors explain why they have defined SGA/LGA in a different way?**

There are different definitions of SGA/LGA in the literature. We have used the definition that is mostly used in Sweden. This is based on ultrasonic estimates of foetal weight in normal pregnancies [Marsál K, Persson PH, Larsen T, Lilja H, Selbing A, Sultan B. Intrauterine growth curves based on ultrasonically estimated foetal weights. Acta Paediatr. 1996 Jul;85(7):843-8. doi: 10.1111/j.1651-2227.1996.tb14164.x. PMID: 8819552]. Using percentiles by gestational age and sex instead will systematically underestimate the magnitude of the growth restriction in infants born preterm. Another issue is which cut-off to use to define growth restriction. We could have used z-score <-1.28 (equivalent to the 10th percentile), but then a great proportion of the children below the cutoff would have been classified as SGA even if they are just normal but small. Using a more strict cut-off (-1.96) we are more likely to detect growth-restricted children.

- **Information regarding child infections was obtained from the National Patient Register. Why did the authors use only the inpatient register and did not use the outpatient register data (that started in 2001)? Moreover, the authors should write in the text that they use inpatient care data, this is reported only in Table 1.**

As stated within the methods under section Perinatal characteristics: *“ … any child infection requiring specialist in-patient care since birth until the age of one year (yes, no)…”,* we limited our analysis to infections recorded in in-patient care registries, as these reflect clinically significant cases requiring hospitalization and are more reliably diagnosed and documented. In contrast, outpatient data were not consistently available throughout the study period.

- **The authors wrote that “Traditional case-control analyses using logistic regression do not allow estimation of time-varying associations” and therefore used Cox regression models to analyze their matched case-control data. However, I disagree with them because in a case-control study you can assess time varying associations, for example the effect of an exposure on childhood cancer risk during the first five years of life, by restricting the analyses to the cases diagnosed during the age period of interest and their matched controls. It is with time-varying covariates/exposures that one should use Cox regression when analyzing case-control data. I am not an expert on using Cox regression on case-control data but from the study below from Statistics in Medicine it seems that the naïve approach (analyzing the case-control data using the standard Cox regression model as if the data were obtained from a cohort study) could lead to an underestimation of the association. I therefore suggest to the authors to re-run the analyses using a conditional logistic regression model, as all the exposures that they have analyzed (perinatal factors) are not time varying.
Reference: Statistics in Medicine (2003) - Evaluation of Cox's model and logistic regression for matched case-control data with time-dependent covariates: a simulation study**

We thank the reviewer for this comment. We are aware that traditionally in case–control settings with matched controls and time-fixed exposures, conditional logistic regression is commonly applied. Cox regression falls under statistical techniques aimed at analysing “time-to-event” data and/or assessing the relationship between a given exposure and the occurrence of an outcome after a follow-up period among a cohort of individuals (ElHafeez et al., 2021; [10.1155/2021/1302811](https://doi.org/10.1155/2021/1302811)). In the present study, follow-up lengths, or time-to-event, vary considerably, with children born in the later part of the study period having significantly shorter follow-up compared to those born in the early years of the study. As such we wanted to address age at diagnosis (follow-up) as a potential factor influencing our tested association. While we agree that such patterns can also be explored through stratified logistic regression (restricting to cases diagnosed within specific age windows), such approaches require arbitrary partitioning of time and substantially reduce statistical power. In contrast, the Cox model allows us to use the full temporal information.

With the design of the present study, using loose matching (year of birth, county of birth and sex only), we don’t think that a matched analysis would be appropriate. In a simulation study, Kuo et al conclude: “Unmatched methods, e.g., unconditional logistic regression, are viable options for loose-matching data based on our findings. When the study design involves other complex features such as censoring and repeated measures, matching on a few demographic variables can be ignored if the confounding effect is not very large. Standard methods such as Cox regression and generalised estimating equation then can be readily applied.” [Kuo CL, Duan Y, Grady J. Unconditional or Conditional Logistic Regression Model for Age-Matched Case-Control Data? Front Public Health. 2018 Mar 2;6:57. doi: 10.3389/fpubh.2018.00057. PMID: 29552553; PMCID: PMC5840200].

Based on the comments from the other two reviewers, who supported our choice of statistical analyses—one specifically highlighting the use of Cox regression as a strength of the study—we have decided to retain Cox regression in the present study, unless the editor advises otherwise.

- **The authors wrote “The models were not assessed for characteristics or categories with fewer than ten observations.” Please clarify whether with “ten observations” you mean ten cancer cases for a specific category or if with ten observations you meant a combination of cases and controls**

We have rephrased the sentence as follows: *“The models were not assessed for characteristics or categories when there were fewer than ten observations in a group of cases or controls.”*

**- Why did the authors add a quadratic term for BMI in the regression models? In the main analysis BMI was used as a categorical variable rather than a continuous one**

Inclusion of the quadratic term of BMI was run as a sensitivity analysis; this was omitted from the manuscript.

**- Did the authors have information regarding genetic syndromes? As these are linked to childhood cancer risk and could also be linked to some perinatal characteristics, is it possible to additionally adjust for genetic syndromes, at least for the most common ones such as Down syndrome, neurofibromatosis type 1, etc.?**

We additionally obtained data on the diagnosis of some cancer predisposing syndromes, including Down syndrome, Neurofibromatosis type 1, Congenital malformation syndromes involving early overgrowth, and Von Hippel-Lindau syndrome. We conducted further analyses assessing the association between the diagnosis of these syndromes (yes/no) and the risk of childhood cancer. As suggested, we also performed a sensitivity analysis evaluating the association of all studied perinatal characteristics after excluding children diagnosed with any of these syndromes (n = 49). The exclusion of those children did not significantly affect our findings. These analyses are now added into the supplementary materials and described within the methods, results and discussion part of the manuscript.

**- In the discussion the authors wrote “However, several studies, including a large cohort with over 11 thousand cases from Sweden, Denmark and Finland, reported null associations (65,66).”. However, references 65 and 66 refer to studies performed in the UK and the US. Please check that all references are cited correctly.**

We have corrected the citation order of the references.

**- I agree with the authors’ decision to not adjust for multiple comparisons. However, the authors should mention in the discussion section that because of the low number of cases and the several associations analyzed,** **it is possible that some of the reported statistically significant associations could be chance findings.**

According to the comments and suggestions of 2 reviewers (reviewer 1 and 2), the following was added into the discussion of the manuscript within the limitations of the study part: *“The statistical analysis of this study includes multiple comparisons, increasing the risk of false positives; however, given the limited statistical power, lowering the significance threshold could potentially overlook meaningful associations. Therefore, we chose to interpret the results cautiously in the context of existing literature rather than adjust for multiple comparisons, acknowledging that some statistically significant associations may be due to chance. “*

**- In the last paragraph of the discussion, the authors wrote that “Nevertheless, as genetic predisposition explains less than 10% of childhood cancer cases (4), we believe that other perinatal factors, such as environmental factors, could contribute to our findings.” The authors also stated in the conclusion paragraph that “The reported results strongly highlight the significance of the perinatal period as a critical window, particularly for early-onset childhood cancer”. Can the authors report and comment on the proportion of childhood cancer cases that are caused by the exposure assessed in the current study?**

In contrast to the above-referred study - reporting proportion of childhood cancers attributable to genetic predisposition based on genome sequencing data - our study is a case–control analysis designed to evaluate associations between perinatal characteristics (i.e. exposures) and childhood cancer risk. Because association studies cannot determine causal effects or quantify the fraction of cases attributable to specific exposures, we are not able to report the proportion of childhood cancers “caused” by the perinatal characteristics we assessed. Any such estimate would require robust evidence of causality—typically from mechanistic studies, randomized interventions, etc.—which is beyond the scope of the current study design.

The statement on perinatal window as a critical window associated with risk of childhood cancer is based on our results, showing significant associations with risk of childhood cancer for several perinatal characteristics, particularly when considering early diagnosed cancer cases; this has been reported also in previous studies (e.g. Bjørge et al. 2013 doi:10.1542/peds.2013-1317 ; Hoang et al., 2024, doi: 10.1002/ijc.34683).

**Moreover, in the abstract the authors wrote “Results indicate several perinatal characteristics associated with the risk of childhood cancer, which is important for prevention”. If the authors believe that these results are important for prevention, this should be thoroughly discussed in the discussion section, together with a discussion on the potential number of cases explained by these exposures and how these results can be used for preventive measures.**

According to the reviewers comment we have revised our conclusion within the abstract as follows: *“Our findings identify several perinatal characteristics associated with childhood cancer risk, highlighting the perinatal period as an important window for future etiological research.”*

**Minor comments:
- Check the spelling used throughout the manuscript as sometimes you write leukemia and other times you write leukaemia**

The manuscript has been proofread by a native speaker, and spelling and grammatical errors have been corrected.

**- Table 2: Why some ICCC-3 groups are written in italic?**

It has been revised (italics removed).

**- Table 2: the columns percentages in some instances are not correct and when summed together gave a total > 100%**

We thank the reviewer for this observation. We have corrected the table.

**- Tables should be presented in the text in numerical order. In the text, Table 3 is mentioned before Table 2**

It has been corrected**.
- Footnote Table 4: did the authors mean to write inpatient register rather than incoming patient register**

This has been revised to match the definition of the registry in the methods of the manuscript, as follows: *“data according to the National Patient Register (in-patient care; since 1990)”*

**- Table 4: why are some numbers underlined in table 4?**

Underlining was removed.

**Reviewer #3:** This is a reasonable study design given the available sample. The major variables of perinatal characteristics, parental education and GA are well formulated as necessary. The variable exclusions for missingness, incompleteness or otherwise made sense.

The statistical analysis section starts on line 165. The investigators give a logical rationale for the approach stating that, ‘Traditional case-control analyses using logistic regression do not allow estimation of time-varying associations. Therefore, in the present study, the Cox proportional hazards regression with child age as the underlying time scale was used to examine the association between perinatal characteristics (Table 1) and risk of childhood cancer.’ Their checking for the appropriateness of proportional hazards was followed appropriately and thus the major model for presenting the results as hazard ratios and their interpretation was reasonable in a somewhat time dependency situation.

-**However, their explanation of the GA as a potential risk factor of childhood cancer and for most of these characteristics the relationships with GA still remains unclear in this context from the statistical perspective. They note that to investigate GA as a potential risk factor while addressing potential collider bias, they conducted three separate models as outlined. Some detail about the process with actual variable examples would be helpful to the reader.**

As suggested, we have added more information on the modelling process, particularly stating variables, within the methods of the manuscript as follows: *”We completed a crude Cox regression model for all perinatal characteristics separately. Subsequently, models with stepwise adjustments were conducted to identify possible influencing variables. The first model was adjusted for all parental characteristics (i.e. maternal cancer diagnosis, maternal and paternal age and education, parity, maternal BMI and maternal smoking), the second additionally to parental also for pregnancy characteristics (i.e. assisted pregnancy with IVF and mode of delivery), and the third, besides parental and pregnancy characteristics, also for GA.”*

**-The Results start on line 207 and series of descriptive tables follow. The tables have so much detail that they are cumbersome to get through especially Tables S1 to S13. –**

**There is no discussion of any confounding influences (since adjustments were made as per the investigators), just a summary list of possibly associative characteristics with the outcome. Where there any competing risks in the process?**

We are aware of the complexity of the tables, particularly those included within S1-S13, which corresponds to the extensive statistical analysis performed within the study. We have tried to simplify the tables within the main text as much as possible; however, the given information within the tables is necessary for the understanding of the results and analysis performed, particularly as we are unable to share our data publicly (due to the restrictions from the ethical board).

Within several parts of the discussion related to specific characteristics (pages 18, 19, 20, 21, 22), we have commented on the potential confounding of other perinatal characteristics with additional support from the literature.

**- The limitations are noted on page 17. The investigators note the low numbers in some categories resulting in low power. Was the geographic coverage too small or initial sampling period too narrow. Where does power come into this situation which is a case control study? How relevant is the county of birth in the matching process or is this tied to a genetic or environmental consideration?**

As described under Study design and population, this study is a part of the FeToxCancer project which aims to clarify the effects of chemical exposure in utero and possible underlying mechanisms in the aetiology of childhood cancer within two phases; where in the first phase we obtained extensive registry data and, in the second, we will add measurements of toxicants in biobanked maternal serum samples obtain during pregnancy. The county of birth in the matching process is therefore tied to considerations of environmental exposure, which might vary between counties. Moreover, as those samples have been stored only in the southern part of Sweden and have been biobanked since 1989, this has determined the time period and the geographic location for the study population. We have also added this latter sentence into the manuscript to make the selection of the population clearer.
